# Supplementary material for: Curated and harmonised transcriptomics datasets of interstitial lung diseases
Source: Data Brief. 2025 Oct 14;63:112139. doi: 10.1016/j.dib.2025.112139 (PMC12581653; doi:10.1016/j.dib.2025.112139)
Supplement: Supplementary file 1 [file mmc1.zip › Supplementary_material/RNA-seq/fastQC reports/GSE99621/TRIMMED_SRR5642422_1_fastqc.html]

TRIMMED\_SRR5642422\_1.fastq.gz FastQC Report 

FastQC Report

Thu 24 Nov 2022  
TRIMMED\_SRR5642422\_1.fastq.gz

## Summary

- Basic Statistics
- Per base sequence quality
- Per tile sequence quality
- Per sequence quality scores
- Per base sequence content
- Per sequence GC content
- Per base N content
- Sequence Length Distribution
- Sequence Duplication Levels
- Overrepresented sequences
- Adapter Content

## Basic Statistics

| Measure | Value |
| --- | --- |
| Filename | TRIMMED\_SRR5642422\_1.fastq.gz |
| File type | Conventional base calls |
| Encoding | Sanger / Illumina 1.9 |
| Total Sequences | 35495459 |
| Sequences flagged as poor quality | 0 |
| Sequence length | 60-126 |
| %GC | 44 |

## Per base sequence quality

## Per tile sequence quality

## Per sequence quality scores

## Per base sequence content

## Per sequence GC content

## Per base N content

## Sequence Length Distribution

## Sequence Duplication Levels

## Overrepresented sequences

| Sequence | Count | Percentage | Possible Source |
| --- | --- | --- | --- |
| AAGCAGTGGTATCAACGCAGAGTACTTTTTTTTTTTTTTTTTTTTTTTTT | 2322833 | 6.54402863194416 | Clontech SMART CDS Primer II A (100% over 26bp) |
| AAGCAGTGGTATCAACGCAGAGTACATGGGGAGGCATTGAGGCAGCCAGC | 45179 | 0.12728106995320163 | Clontech SMARTer II A Oligonucleotide (100% over 25bp) |
| AAGCAGTGGTATCAACGCAGAGTACATGGGAAGCAGTGGTATCAACGCAG | 38798 | 0.10930412253578689 | Clontech SMARTer II A Oligonucleotide (100% over 25bp) |

## Adapter Content

Produced by FastQC (version 0.11.7)
